# High mutation detection rates in cerebral cavernous malformation upon stringent inclusion criteria: one-third of probands are minors

**DOI:** 10.1002/mgg3.60

**Published:** 2014-01-14

**Authors:** Stefanie Spiegler, Juliane Najm, Jian Liu, Stephanie Gkalympoudis, Winnie Schröder, Guntram Borck, Knut Brockmann, Miriam Elbracht, Christine Fauth, Andreas Ferbert, Leonie Freudenberg, Ute Grasshoff, Yorck Hellenbroich, Wolfram Henn, Sabine Hoffjan, Irina Hüning, G Christoph Korenke, Peter M Kroisel, Erdmute Kunstmann, Martina Mair, Susanne Munk-Schulenburg, Omid Nikoubashman, Silke Pauli, Sabine Rudnik-Schöneborn, Irene Sudholt, Ulrich Sure, Sigrid Tinschert, Michaela Wiednig, Barbara Zoll, Mark H Ginsberg, Ute Felbor

**Affiliations:** 1 Department of Human Genetics, University Medicine Greifswald and Interfaculty Institute of Genetics and Functional Genomics, University of GreifswaldGreifswald, Germany; 2 Department of Medicine, University of California San DiegoSan Diego, California; 3 Institute of Human Genetics, University of UlmUlm, Germany; 4 Department of Paediatrics and Paediatric Neurology, University of GöttingenGöttingen, Germany; 5 Institute of Human Genetics, University of AachenAachen, Germany; 6 Division of Human Genetics, Medical University InnsbruckInnsbruck, Austria; 7 Department of Neurology, Klinikum Kassel GmbHKassel, Germany; 8 Department of Neuropaediatrics, University Hospital DresdenDresden, Germany; 9 Institute of Medical Genetics and Applied Genomics, Rare Disease Center Tübingen, University of TübingenTübingen, Germany; 10 Institute of Human Genetics, University of LübeckLübeck, Germany; 11 Department of Human Genetics, Saarland UniversityHomburg/Saar, Germany; 12 Department of Human Genetics, Ruhr-UniversityBochum, Germany; 13 Department of Neuropaediatrics, Children's HospitalOldenburg, Germany; 14 Institute of Human Genetics, Medical University GrazGraz, Austria; 15 Institute of Human Genetics, University of WürzburgWürzburg, Germany; 16 Institute of Human Genetics, University of FreiburgFreiburg, Germany; 17 Department for Interventional and Diagnostic Neuroradiology, University Hospital AachenAachen, Germany; 18 Institute of Human Genetics, University of GöttingenGöttingen, Germany; 19 Institute of Medical Genetics, University of ZürichZürich, Switzerland; 20 Department of Neurosurgery, University Hospital EssenEssen, Germany; 21 Institute of Clinical Genetics, Technical University of DresdenDresden, Germany; 22 Department of Environmental Dermatology and Venereology, Medical University GrazGraz, Austria

**Keywords:** Age at disease onset, *CCM1*, *CCM2*, *CCM3*, cerebral cavernous malformation, HEG1, mutation detection rate, predictive testing

## Abstract

Cerebral cavernous malformations (CCM) are prevalent vascular malformations occurring in familial autosomal dominantly inherited or isolated forms. Once CCM are diagnosed by magnetic resonance imaging, the indication for genetic testing requires either a positive family history of cavernous lesions or clinical symptoms such as chronic headaches, epilepsy, neurological deficits, and hemorrhagic stroke or the occurrence of multiple lesions in an isolated case. Following these inclusion criteria, the mutation detection rates in a consecutive series of 105 probands were 87% for familial and 57% for isolated cases. Thirty-one novel mutations were identified with a slight shift towards proportionally more *CCM3* mutations carriers than previously published (*CCM1*: 60%, *CCM2*: 18%, *CCM3*: 22%). In-frame deletions and exonic missense variants requiring functional analyses to establish their pathogenicity were rare: An in-frame deletion within the C-terminal FERM domain of CCM1 resulted in decreased protein expression and impaired binding to the transmembrane protein heart of glass (HEG1). Notably, 20% of index cases carrying a *CCM* mutation were below age 10 and 33% below age 18 when referred for genetic testing. Since fulminant disease courses during the first years of life were observed in *CCM1* and *CCM3* mutation carriers, predictive testing of minor siblings became an issue.

## Introduction

Cerebral cavernous malformations (CCM; MIM 116860, 603284, 603285) are autosomal dominantly inherited vascular malformations mainly caused by unambiguously pathogenic heterozygous loss-of-function mutations in one of three genes, *CCM1*,*CCM2* or *CCM3*. In 2008, we had presented a first follow-up on 28 probands affected with CCM (Stahl et al. 2008). The mutation detection rates had been extremely high, being 94% for familial CCM and 60% for isolated CCM. Since 2005, inclusion criteria for diagnostic genetic testing have either been a positive family history or the occurrence of multiple lesions in an isolated case. Of note, our molecular genetic testing had been based on direct sequencing of *CCM1*,*CCM2*, and *CCM3*, quantitative analyses to detect larger *CCM1-3* deletions/duplications using multiplex ligation-dependent probe amplifications (MLPA), and – when required – transcript, protein expression, and interaction analyses from the very beginning onwards.

We here present a consecutive series of 77 further index cases analyzed since 2009 and highlight that the proportion of children and adolescents under the age of 18 presenting as index cases is higher than previously thought (Gunel et al. 1996; Siegel et al. 2005). In our cohort, one-third of index patients that were shown to carry a heterozygous mutation in either *CCM1*,*CCM2* or *CCM3* are children or adolescents. Since fulminant courses of the disease have only rarely been reported in infants affected with CCM (Ng et al. 2006; Sürüçü et al. 2006; Gianfrancesco et al. 2007), we describe two *CCM1* mutation carriers who presented with hemiparesis during their second and third year of life requiring immediate surgical intervention. One individual's younger sibling had predictive genetic testing several years later.

## Materials and Methods

### Genetic testing

Genotype-phenotype analyses of individuals affected with CCM were approved by local ethics committees (University of Würzburg, Study 21/05, University Medicine Greifswald, No. BB 94/11a) and performed with informed consent. Genomic DNA was extracted from peripheral blood leukocytes and all coding *CCM1-3* exons and adjacent splice sites were directly sequenced on an ABI 3130xl automated sequencer (Applied Biosystems, Life Technologies GmbH, Darmstadt, Germany) and analyzed with SeqPilot software (JSI medical systems GmbH, Kippenheim, Germany). Mutation-negative individuals were subsequently screened for large *CCM1-3* alterations using SALSA MLPA Kits P130 & P131 (MRC Holland, Amsterdam, Netherlands) (Gaetzner et al. 2007). GenBank and Ensembl accession numbers are as follows: *CCM1* (GenBank: NM_194456.1, Ensembl: ENST00000394507; numbering of coding exons 5-20), *CCM2* (GenBank: NM_031443.3, Ensembl: ENST00000258781), and *CCM3* (GenBank: NM_145860.1, Ensembl: ENST00000392750; numbering of coding exons 4-10). Sequences were analyzed in SeqPilot with the Ensembl datasets. DNA mutation numbering is based on cDNA sequence with +1 corresponding to the A of the ATG translation initiation codon. In silico analyses to predict the pathogenicity of unclassified variants were performed using MutationTaster (http://www.mutationtaster.org/), MutPred (http://mutpred.mutdb.org/), PolyPhen-2 (http://genetics.bwh.harvard.edu/pph2/), and SIFT (http://sift.jcvi.org/www/SIFT_enst_submit.html) for the exonic missense and small in-frame mutations and BDGP (http://www.fruitfly.org/seq_tools/splice.html), Human Splicing Finder (http://www.umd.be/HSF/), and NetGene2 (http://www.cbs.dtu.dk/services/NetGene2/) for mutations affecting splice sites and c.313G>C.

### Transcript analyses

In order to analyze the effects of the *CCM1:c.2025+1G>A* splice site mutation, RNA was extracted from untreated peripheral blood leukocytes using the PAXgene Blood RNA kit (PreAnalytiX, Qiagen, Hilden, Germany). cDNA was synthesized using SuperScript™ III Reverse Transcriptase (Invitrogen, Life Technologies GmbH, Darmstadt, Germany). The region around the presumed skipping of exon 18 of *CCM1* was amplified using a cDNA-specific forward primer complementary to the exon 16/exon 17 junction (5′-AGCAAGGTTTCCTAAATGAAG-3′) and a specific reverse primer complementary to the exon 19/exon 20 junction (5′-CGAGACCAGCCTGTTTTGTA-3′). PCR products were size-separated by agarose gel electrophoresis prior to TOPO TA-Cloning (Invitrogen) and sequencing.

### Generation of plasmids

The human full-length FLAG-tagged *CCM1* (FLAG-CCM1) construct used for transient expression in HEK293 cells has been described previously (Stahl et al. 2008). To generate FLAG-CCM1:p.N607_K675del, the altered coding sequence of *CCM1* lacking human exon 18 (*CCM1:c.2025+1G>A*, CCM1:p.N607_K675del) was similarly fused to a FLAG tag, PCR-cloned into the pCR2.1 vector and subcloned into pcDNA3.1(+) vector. The plasmid pRc/CMV2-EGFP served as positive control for transfections and as negative control for western blots.

### Protein expression and HEG1-binding assay

HEK293A cells were cultured in DMEM (MediaTek) supplemented with 10% FBS (Sigma, Saint Louis, MO), l-glutamine, nonessential amino acids, and penicillin/streptomycin (all from Invitrogen). Cells were transfected with Lipofectamine Plus (Invitrogen) reagent following the manufacturer's protocol. Twenty-four hours after transfection, cells were washed twice with ice-cold PBS and lysed in ice-cold lysis buffer (50 mmol/L Tris-HCL pH 7.4, 150 mmol/L NaCl, 0.5% NP-40, 5 mmol/L MgCl_2_) with freshly supplemented EDTA-free protease inhibitors (Roche, Mannheim, Germany). Cell lysates were cleared by centrifuging at 20,000*g* for 15 min at 4°C. An aliquot of supernatant was boiled in SDS-PAGE sample buffer and resolved by SDS-PAGE. After transferring onto a nitrocellulose membrane, western blot was performed using mouse anti-FLAG M2 antibody (Sigma) to determine the FLAG-tagged CCM1 protein expressions.

The final lysate volumes were adjusted to level the expression of FLAG-CCM1 wild-type and mutant proteins. Recombinant biotinylated HEG1 and integrin *α*II*β* cytoplasmic tail protein production was described previously (Kleaveland et al. 2009). ELISA plates were coated with 50 *μ*L of 10 *μ*g/mL neutravidin (Thermo, Schwerte, Germany) for overnight, blocked with 1% BSA (Sigma) and then incubated with 2 *μ*g/mL HEG1 tail protein or *α*II*β* tail protein, respectively. After washing three times with wash buffer (0.2% Triton X-100, 1× PBS), plates were incubated with cell lysates accordingly. After washing, the plates were incubated with mouse anti-FLAG M2 antibody (Sigma) and subsequently rabbit anti-mouse IgG HRP-conjugated antibody (Thermo). The plates were developed with chemiluminescence reagent (Thermo) and read using Victor 2 plate reader (PerkinElmer, Waltham, MA). Data were analyzed in Graphpad Prizm software.

## Results

Summarizing the data published by Stahl et al. (2008; 28 probands divided into 23 mutation carriers and five mutation-negative individuals, see Table S1) and those derived from the current series of 77 further probands (Table 1; including 56 mutation carriers, two unclassified variants and 19 mutation-negative individuals), 79 of the 105 index cases were found to carry a heterozygous mutation in either the *CCM1*, the *CCM2* or the *CCM3* gene. 60% of mutations were found in the *CCM1* gene (*n* = 48), 18% in *CCM2* (*n* = 14), and 22% in *CCM3* (*n* = 17). 32% (*n* = 25) of index cases had a stop mutation, 34% (*n* = 27) a frameshift mutation, 28% (*n* = 22) a splice site mutation, affecting or located next to a consensus AG/GT splice site and predicted to result in a frameshift, and 4% (*n* = 3) a large genomic deletion. In two cases (2%), a splice site mutation resulted in single exon in-frame deletions of *CCM1* exon 18 and *CCM1* exon 19, respectively. A three base pair in-frame deletion within *CCM1* in an isolated case and an exonic *CCM1* missense mutation were categorized as unclassified variants (UV) and are highlighted by bold letters in Table 1. In its last column, Table 1 also demonstrates that one-third of index cases carrying a *CCM* mutation were below age 18 when referred for genetic testing.

**Table 1:** *CCM1*–*3* mutations and UVs in the current series of 77 consecutive, unrelated probands.

Gene	Exon^1^	Nucleotide exchange	Predicted amino acid change	Presentation^2^	Reference	Age at referral to genetic testing
*CCM1*	6	c.143dupA	p.R49Efs^*^15	f	Cavé-Riant et al. (2002)	51
	**7**	**c.313G>C**	**p.G105R**	**f**	**Novel**	**12**
	8	c.406C>T	p.Q136^*^	f	Novel	46
	9	c.557dupT	p.T188Nfs^*^2	f	Novel	1
	IVS9	c.729+5G>A	p.Q201Rfs^*^2^3^	f	Novel	44
	IVS9	c.730-1G>A	p.?	i	Davenport et al. (2001)	27
	10	c.842delA	p.D281Afs^*^7	f	Novel	28
	IVS10	c.845+1G>A	p.?	i	Novel	36
	IVS10	c.845+1G>T	p.?	f	Novel	77
	11	c.877delC	p.H293Tfs^*^12	f	Novel	15
	12	c.1093G>T	p.G365^*^	f	Novel	1
	13	c.1166delG	p.G389Efs^*^5	f	Novel	71
	**13**	**c.1197_1199delCAA**	**p.N399del**	**f**	**Novel**	**44**
	IVS13	c.1255-1G>A	p.Y419Ffs^*^15^3^	f	Cavé-Riant et al. (2002)	31
	14	c.1255_1270delTATGAAAAAGTTCGAA	p.E420Tfs^*^12	f	Novel	78
	14	c.1267C>T^4^	p.R423^*^	i/f	Cavé-Riant et al. (2002)	23,63,79,44,32
	14	c.1306_1310delTTGAA	p.L436Afs^*^4	f	Battistini et al. (2007)	7
	IVS14	c.1412-1G>C	p.?	i	Novel	26
	15	c.1431T>A	p.Y477^*^	i	Novel	24
	15	c.1531delG	p.D511Mfs^*^2	i	Novel	23
	IVS15	c.1564-1G>A	p.?	f	Novel	32
	16	c.1619T>A	p.L540^*^	f	Novel	10
	16	c.1660_1678delTTGGCAAGTCTGCTTTTGC	p.L554Kfs^*^2	i	Novel	14
	16	c.1678C>T	p.Q560^*^	f	Stahl et al. (2008)	8
	IVS16	c.1730+4_1730+7delAGTA^4^	p.I522^*^^3^	f	Cavé-Riant et al. (2002)	51,17
	17	c.1806_1807insAT	p.H603Ifs^*^59	i	Novel	31
	18	c.1950dupC	p.S651Qfs^*^4	f	Novel	13
	18	c.1961_1962delAA	p.K654Sfs^*^21	f	Novel	43
	IVS18	c.2025+1G>A	p.N607_K675del^3^	f	Denier et al. (2004)	21
	IVS18	c.2026-12A>G	p.A676_Q714del^3^	f	Laberge-le Couteulx et al. (1999), Cavé-Riant et al. (2002)	5
		Deletion of entire *CCM1* gene		f	Gaetzner et al. (2007)	51
*CCM2*	1	c.1-36_4del, 40 bp deletion including start codon	p.?	i	Novel	35
	1	c.30G>A^4^	No transcript	i/f	Liquori et al. (2003)	16,71
	IVS2	c.205-2_205-1delAGinsT	p.Y69Vfs^*^3^3^	f	Stahl et al. (2008)	43
	10	c.1071_1074dupCCCT	p.E359Pfs^*^2	f	Novel	47
	10	c.1255dupG	p.D419Gfs^*^2	f	Novel	33
*CCM3*	4	c.63_64dupCC	p.L22Pfs^*^13	f	Novel	42
	5	c.103C>T^4^	p.R35^*^	i/f	Bergametti et al. (2005)	8,11,20,1
	5	c.113delT	p.L38Rfs^*^7	f	Novel	53
	IVS6	c.269-1G>T	p.?	f	Novel	3
	7	c.317delA	p.K106Rfs^*^20	i	Novel	2
	7	c.334_337delCAAA	p.Q112Ffs^*^13	f	Novel	20
	7	c.391delA	p.I131Sfs^*^4	f	Novel	1
	IVS8	c.475-2A>G	p.A119Gfs^*^42^3^	i	Novel	22
	IVS9	c.558-1G>C^4^	p.?	i/f	Novel	11,36
	IVS9	c.558-2A>C	p.?	i	Novel	3
	10	c.586C>T	p.R196^*^	i	Bergametti et al. (2005)	17
	10	c.598C>T	p.Q200^*^	f	Schröder et al. (2014)	50

UVs are depicted in bold letters. The 19 mutation-negative index cases of this cohort are not included.

1 Exon numbering according to Ensembl *CCM1* transcript NM_194456.1 (coding Exons 5-20), *CCM2* transcript NM_031443.3 and CCM3 transcript NM_145860.1 (coding Exons 4–10).

2 Presentation: f, familial; i, isolated.

3 Confirmed by transcript analysis.

4 Was observed in two or more independent families as judged by family history, ages are given for all index patients.

### Case report of a 1-year-old *CCM1* mutation carrier and predictive testing of his infant sister

**Figure 1:**
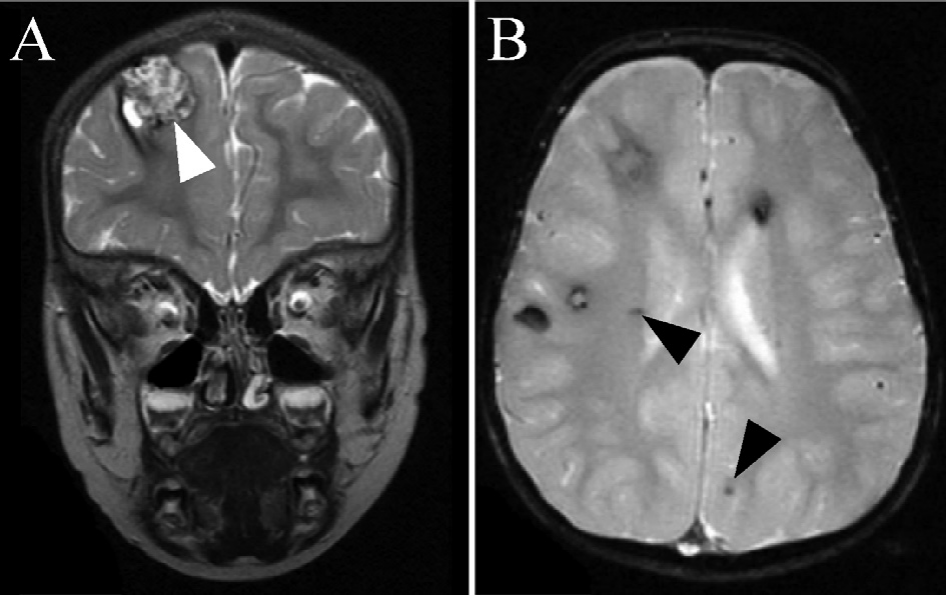
Characteristic MRI findings in CCM. MRI of a 1-year-old *CCM1* mutation carrier showing coronal (A) T2 and (B) axial T2*w sequences. (A) There is a huge mulberry-like cavernoma in the right frontal lobe (white arrowhead). Blood-degradation products of varying age cause a reticulated signal within the cavernoma. (B) T2*w imaging reveals multiple hypointense areas corresponding to hemosiderin depositions of several larger and dot-sized cavernomas (arrowheads).

As an example for fulminant disease courses in infants, we here depict in more detail an index case that had experienced progressive right-sided hemiparesis at the age of 18 months prior to resection of a hemorrhaging cavernoma in the left cerebral hemisphere. At that time, a further larger cavernoma had been observed in the right frontal lobe, which increased in size and was electively resected at the age of 2 years and 4 months (Fig. 1A). In addition, the boy revealed multiple further silent cavernomas (Fig. 1B) and a vascular retinal malformation. At the age of 3½ years, mild residual hemiparesis primarily of the right leg was documented, while yearly MRI demonstrated stable cavernomatosis until the age of currently 8 years. The proband was already listed in the cohort from Stahl et al. 2008 and is heterozygous for the *CCM1* gene mutation c.1255-4_1255-2delGTA, which is predicted to destroy the splice acceptor site of exon 14 and to result in a truncated protein. This mutation had also been reported to segregate in a three-generation pedigree (Verlaan et al. 2002).

The proband's father reported that he had experienced headaches in his childhood, but has been asymptomatic thereafter. The parents are first degree cousins and both parents decided to be genetically tested and actively asked for predictive testing of their younger daughter when she was 3 years and 8 months old. Only the father, but not his daughter turned out to carry his son's mutation, but thus far has not decided to ask for neuroimaging.

### Functional characterization of a single exon in-frame deletion within the FERM domain of CCM1: decreased protein expression and loss of binding to HEG1

Previously, a genomic deletion involving *CCM2* exon 2 had been shown to lead to an in-frame deletion of 58 amino acids resulting in impaired binding of CCM2:p.P11_K68del to CCM1 and the inability to form a CCM1/CCM2:p.P11_K68del/CCM3 complex in vitro (Stahl et al. 2008). An in-frame deletion of 18 amino acids encoded by *CCM3* exon 5 (CCM3:p.L33_K50del) had been reported to result in loss of CCM3:p.L33_K50del binding to serine/threonine protein kinases STK25 and MST4 (Voss et al. 2009). Therefore, we intended to elucidate the mechanisms of action of CCM1:p.N607_K675del identified in this study.

**Figure 2:**
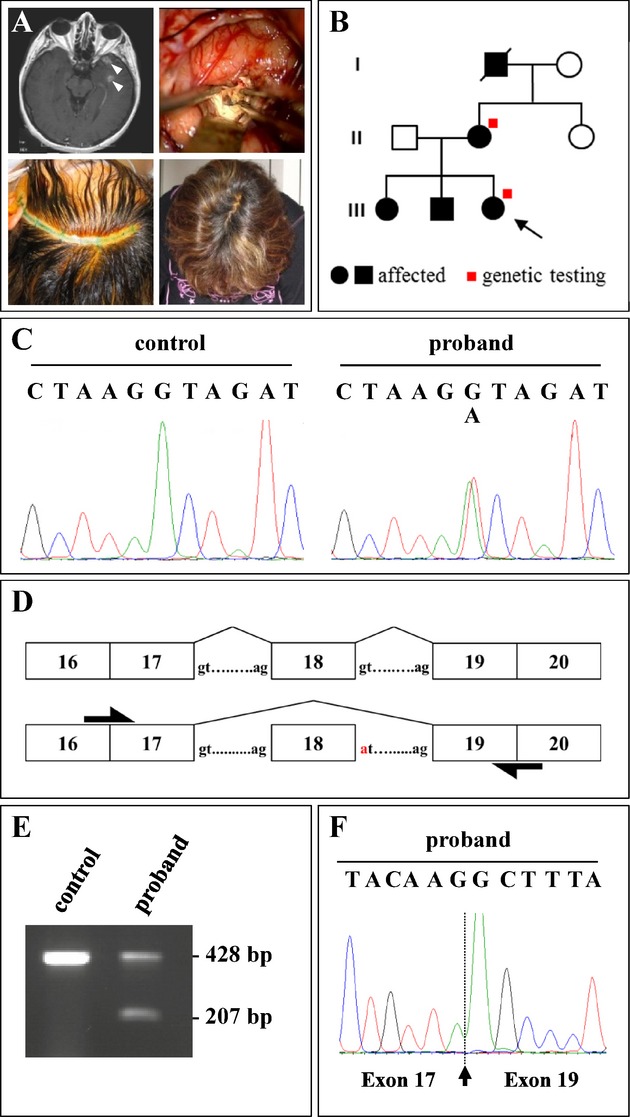
Single exon in-frame deletion within the FERM domain of CCM1. (A) Contrast-enhanced T1-weighted MR showing two left temporal cavernomas (white arrowheads) causing drug-resistant epilepsy in a 46-year-old female (upper left panel). Intraoperative view after resection of the two temporal cavernomas and their surrounding hemosiderin (upper right panel). Minimal skin incision (lower left panel). Cosmetic result after 1 week (lower right panel). (B) Pedigree of the proband's family. (C and D) Direct sequencing revealed the splice site mutation c.2025+1G>A within *CCM1* likely resulting in skipping of exon 18. (E) A smaller transcript is visible in the proband. (F) Electropherogram showing the junction between exon 17 and exon 19 in the smaller transcript.

The mutation c.2025+1G>A within *CCM1* (CCM1:p.N607_K675del) was found in a 21-year-old female who reported to have experienced sudden right-sided hemiparesis at the age of 2 years. A large cavernoma in the left basal ganglia had only been partially removed and her right-sided hemiparesis persisted. Her mother suffered from drug-resistant epilepsy due to multiple cavernomas since the age of 42 and underwent surgical resection of two temporal cavernomas at the age of 46 (Fig. 2A). She also turned out to be a heterozygous carrier of *CCM1:c.2025+1G>A* (Fig. 2B and C).

*CCM1:c.2025+1G>A* affects a canonical splice site and is predicted to lead to an in-frame deletion of exon 18 (Fig. 2D). Screening of the entire *CCM1*,*CCM2*, and *CCM3* genes by direct sequencing and MLPA did not demonstrate a further pathogenic mutation in this proband. Consequently, cDNA analyses were performed demonstrating aberrant splicing of exon 18 resulting in a *CCM1* transcript lacking exon 18 (Fig. 2E and F). *CCM1* Exons 14 to 20 encode for the C-terminal FERM domain of the CCM1/KRIT1 protein. Numerous mutations of all types have been reported to cluster within this region. In particular, independent transcript analyses have shown that the splice site mutation *CCM1:c.2026-12A>G* results in the deletion of entire exon 19, which encodes 39 amino acids (CCM1:p.A676_Q714del) (Table 1 and Laberge-le Couteulx et al. 1999; Cavé-Riant et al. 2002; Riant et al. 2013b).

**Figure 3:**
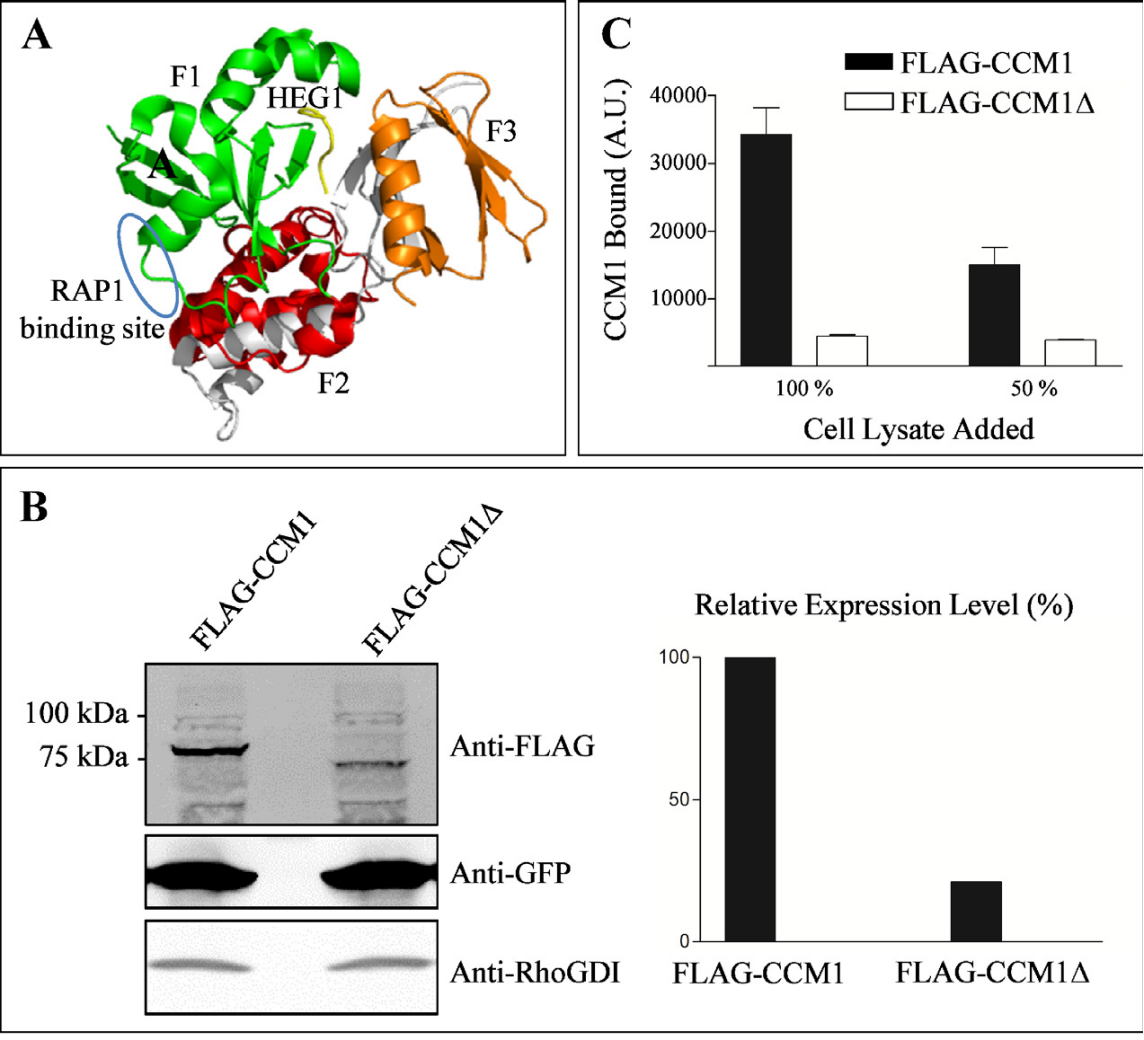
Single exon in-frame deletion within the FERM domain of CCM1 results in decreased protein expression and abrogates binding to HEG1. (A) Molecular view of CCM1 FERM domain bound to the HEG1 tail. The HEG1 tail is shown in yellow. The CCM1 FERM domain consists of three subdomains: F1 (green), F2 (red), and F3 (orange). The deletion in CCM1 encompassing the amino acids encoded by exon 18 is shown in gray and results in the deletion of structural elements within the FERM subdomains F2 and F3. The model also illustrates that the deletion spares the RAP1-binding site within CCM1 which is encircled in blue. (B) Western blot analysis of protein lysates. Compared with wild-type 84 kDa FLAG-CCM1 (lane 1), FLAG-CCM1:p.N607_K675del (FLAG-CCM1Δ) migrated at about 77 kDa (lane 2) and its expression was significantly reduced to ∼20% of the FLAG-CCM1 wildtype, while cotransfected GFP and endogenous RhoDGI levels were not altered. (C) Capture ELISA assays demonstrate that HEG1-bound significantly reduced amounts of FLAG-CCM1Δ (open bars) when compared to FLAG-CCM1 (black bars). Cell lysates were adjusted to match the FLAG-CCM1 expression levels.

*CCM1:c.2025+1G>A* is predicted to cause a 69 amino acid deletion (CCM1:p.N607_K675del), which resides within the C-terminal FERM domain of the CCM1/KRIT1 protein that has been shown to interact with several proteins including RAS-related protein RAP1, which was shown to maintain the integrity of endothelial junctions (Glading et al. 2007). Most recently, the structure of the complex of CCM1 FERM domains and the C-terminus of the transmembrane protein heart of glass (HEG1) was solved (Gingras et al. 2012). As demonstrated in a model of the FERM domain (Fig. 3A), the N-terminal beta sheet of the F3 lobe that forms an essential part of the HEG1-binding site is deleted in CCM1:p.N607_K675del, while the major RAP1-binding site is spared. In order to investigate whether such a structurally altered protein would be stable, the expression of recombinant mutant CCM1:p.N607_K675del protein was analyzed. Recombinant FLAG-tagged CCM1 protein lacking the 69 amino acids encoded by exon 18 (FLAG-CCM1:p.N607_K675del) was expressed with significantly reduced efficiency (∼20%) in HEK293 cells when compared to FLAG-tagged CCM1 (Fig. 3B). To analyze whether interaction of CCM1:p.N607_K675del with HEG1 is abrogated, cell lysates were adjusted accordingly to make sure that the abundance of both wild-type and mutant proteins were similar. Capture ELISA assays revealed that FLAG-CCM1:p.N607_K675del showed markedly reduced binding to the HEG1 cytoplasmic tail when compared to FLAG-tagged CCM1 protein indicating that loss of exon 18 disrupts the interaction capacity of CCM1 to HEG1 (Fig. 3C). Thus, the in-frame deletion of exon 18 within *CCM1* fits into the spectrum of loss-of-function mutations observed for *CCM1*,*CCM2*, and *CCM3*.

### Unclassified variants within *CCM1*

The small in-frame deletion c.1197_1199delCAA (CCM1:p.N399del) results in the omission of a conserved asparagine within the ankyrin 4 repeat domain. According to Mutation Taster and SIFT, this deletion is probably damaging. It was found in a 42-year-old individual who had been diagnosed as having multiple cavernomas after a severe attack of headaches. However, this in-frame deletion was inherited from his mother who was asymptomatic and had no lesions on MRI at the age of 70 years. Further in silico analyses revealed no differences in potential splice sites (HSF, NetGene2, BDGP). Taken together, c.1197_1199delCAA was classified as UV.

The novel UV c.313G>C within the *CCM1* gene was found in a 12-year-old proband whose family did not consent to transcript and segregation analyses so far. In silico analyses suggest that this exonic missense variant changes a conserved glycine into an arginine (p.G105R) and is probably damaging (Polyphen2, Mutation Taster, SIFT, MutPred). According to HSF, the use of an alternative downstream splice acceptor within exon 7 is also conceivable. However, *CCM1:c.313G>C* cannot be considered to be a disease-causing mutation at this stage. The patient's parents have been informed that neuroradiological imaging is currently recommended to those at-risk family members who want to specify their disease risk.

## Discussion

Taken together, mutation detection rates for all 105 index cases analyzed between 2005 and 2012 are 87% for familial (*n* = 55 of 63) and 57% for isolated cases (*n* = 24 of 42). Thus, they are slightly lower but consistent with the ones reported previously (Denier et al. 2006; Stahl et al. 2008). Given that Knudson's two-hit hypothesis has been confirmed for several hereditary vascular malformations (Limaye et al. 2009), one explanation of the observed lower mutation detection rate in isolated cases would be the postzygotic occurrence of both hits that may remain undetected in peripheral blood cells with the mutation scanning techniques applied. Notably, the CCM cohort presented in this study was initiated in 2005 in a combined diagnostic and research setting using stringent inclusion criteria. A recent *CCM1-3* gene analysis which included patients referred for cerebral hemorrhages of unknown etiology yielded a significantly lower mutation detection rate of 44% (Riant et al. 2013b).

**Figure 4:**
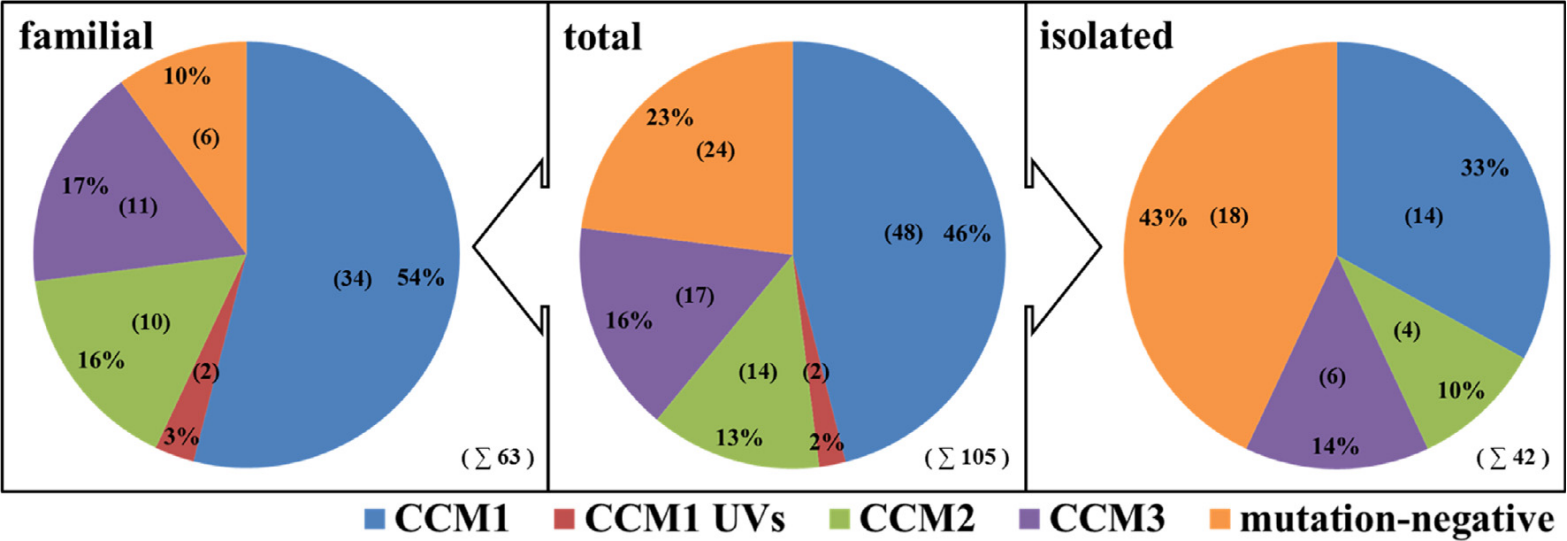
Distribution of mutations of the entire CCM cohort. The data reported by Stahl et al. (2008) are included. Total numbers are given in brackets. UV, unclassified variant, integrated into the figure as a separate entity.

13% (*n* = 8) of familial and 43% (*n* = 18) of isolated cases of this cohort remain mutation-negative (Fig. 4), if the two carriers of the unclassified variants (UV, only 2%) are not considered as pathogenic. Although some of the mutation-negative cases may bear a mutation in regulatory regions of the known *CCM1-3* genes, this observation alludes to further genetic heterogeneity in CCM. However, the identification of novel *CCM* genes using trio-based exome sequencing may be hampered by the above-mentioned postzygotic somatic mosaicism in isolated mutation-negative cases, genetic heterogeneity itself, and incomplete disease penetrance. Magnetic resonance imaging of parents from isolated cases revealed that a large proportion of presumed isolated cases are in fact unrecognized familiar forms (Labauge et al. 1998). Therefore, we suggested to the referring physicians that genetic counseling and molecular as well as neuroradiological evaluation should be offered to parents and subsequently to further at-risk family members of all isolated cases with multiple lesions. Most relatives of individuals categorized as isolated in this study have not yet decided to pursue further steps.

Regarding the distribution of *CCM1*,*CCM2*, and *CCM3* mutation rates, a slight shift toward proportionally more *CCM3* mutations carriers than previously published (Stahl et al. 2008) was observed: 60% of mutations were found in the *CCM1* gene (*n* = 48), 18% in *CCM2* (*n* = 14), and 22% in *CCM3* (*n* = 17). Such a tendency can also be observed in the French cohorts: *CCM3* mutation carriers were found in 13% in 2006, which increased to 16% and 18% in 2013 (Denier et al. 2006; Riant et al. 2013a,b2013b), the distribution being comparable with the cohort presented here: 63% for *CCM1*, 19% for *CCM2*, and 18% for *CCM3* (Riant et al. 2013b). Furthermore, it was suggested in the larger French cohort that *CCM3* mutations might be associated with the development of multiple meningiomas (Riant et al. 2013a): seven unrelated *CCM3* mutation carriers out of 54 were shown to have dural-based lesions with typical radiological features of meningiomas. In line with this observation, two *CCM3* mutations were associated with meningiomas in our group of 17 *CCM3* mutation carriers: c.317delA (unpublished data) and c.598C>T (Schröder et al. 2014).

Notably, the mean age at referral was 18 years for index patients with a *CCM3* mutation (ranging from 1 to 53 years), while the mean age at referral was 30.5 years for *CCM1* probands (ranging from 1 to 79 years) and 40 years for *CCM2* probands (ranging from 16 to 71 years) (Table 1 and Table S1 adding the age at referral to the probands published by Stahl et al. (2008), see Table S1).

Since predictive testing of infant at-risk relatives becomes an issue, we have grouped children and adolescents into two groups, those below age 10 who might require sedation for MRI and those below age 18. In our cohort of 79 index patients who harbored a mutation in *CCM1*,*CCM2*, or *CCM3*, 20% (*n* = 16) of index cases were below the age of 10 and 33% (*n* = 26) of index cases below the age of 18. *CCM1* mutations were identified in 15 probands younger than 18 years (19%) and *CCM3* mutations in 10 (13%), while a *CCM2* mutation was observed only once in this age group in our cohort (1%). Given that the age of disease manifestation (e.g., 2 years for the proband carrying *CCM1:c.2025+1G>A*; Fig. 2) has previously been much earlier than the age at referral to genetic testing (e.g., 21 years for the same proband), disease manifestation is likely much earlier than previously reported.

In the past, it has been referenced that 9% of individuals become symptomatic before age 10 (Siegel et al. 2005). However, the original publication (Gunel et al. 1996) referred to by Siegel et al. (2005) was likely biased toward *CCM1* mutation carriers: Among 47 patients mostly derived from Hispanic American kindreds that later turned out to carry a nonsense founder mutation in *CCM1* (Sahoo et al. 1999; Zhang et al. 2000), only four were given a diagnosis before the age of 10. More recently, mutation carriers have been grouped in two categories, those with disease manifestation before and after age 15 (Denier et al. 2006). In this cohort, 20% of patients became symptomatic before age 15, the proportions being 17% for *CCM1*, 19% for *CCM2*, and 50% for *CCM3* (Denier et al. 2006). The proportions of our patients with an onset below age 15 are 25% for *CCM1* (12/48), 0% for *CCM2* and 53% for *CCM3* (9/17). One explanation for earlier disease manifestation in *CCM3* mutation carriers has been the observation that children harboring a *CCM3* mutation present with significantly more lesions than those with a *CCM1* mutation at a similar age (Nikoubashman et al. 2013).

Predictive testing of at-risk family members below age 18 was requested and performed in 12 cases after thorough genetic counseling (for further information on counseling in the context of predictive testing for CCM see Schröder et al. 2014). As demonstrated for the family of the *CCM1:c.1255-4_1255-2delGTA* mutation carrier who had experienced increasing right-sided hemiparesis at the age of 18 months (Fig. 1A and B), the respective familial mutation was excluded in eight relatives under the age of 18 (data not shown), thus relieving these relatives and their families of unnecessary anxieties and medical examinations.

Improved neuroimaging methods have led to increased identification of incidental isolated cavernomas that often remain clinically silent. Similarly, it can be anticipated that entire *CCM* gene deletions or small mutations within known *CCM* genes will occur as coincidental findings during diagnostic genome-wide array or next-generation sequencing analyses of, for example, syndromic neonates or infants with developmental delay. Better knowledge of early disease manifestations and courses resulting in guidelines on neuropediatric and neuroradiological monitoring and management of mutation carriers in this age group will be useful for genetic counseling of their families.

## Supporting Information

Table S1 Age at referral to genetic testing of 23 probands carrying a *CCM* mutation published by Stahl et al.(2008).
