# Supplementary material for: High mutation detection rates in cerebral cavernous malformation upon stringent inclusion criteria: one-third of probands are minors
Source: Mol Genet Genomic Med. 2014 Jan 14;2(2):176–85. doi: 10.1002/mgg3.60 (PMC3960060; doi:10.1002/mgg3.60)
Supplement: Table S1 — Age at referral to genetic testing of 23 probands carrying a CCM mutation published by Stahl et al.(2008). [file mgg30002-0176-sd1.docx]

| No. | Gene | Nucleotide exchange | Age at referral to genetic testing |
| --- | --- | --- | --- |
| 1 | *CCM1* | Deletion of entire *CCM1* gene | 8 |
| 2 |  | c.1066_1070dupAGTTC | 40 |
| 3 |  | c.1201_1204delCAAA | 18 |
| 4 |  | c.1246_1249delAACA | 25 |
| 5 |  | c.1253delC | 21 |
| 6 |  | c.1255-4_1255-2delGTA | 2 |
| 7 |  | c.1258G>T | 42 |
| 8 |  | c.1678C>T | 51 |
| 9 |  | c.1683_1684insA | 3 |
| 10 |  | c.1710_1711insA | 26 |
| 11 |  | c.1780_1783delGCACinsTACCTGTTACCAAA | 2 |
| 12 |  | c.1815C>G | 41 |
| 13 |  | c.1877T>A | 45 |
| 14 |  | c.2143-2A>G | 15 |
| 15 | *CCM2* | c.30+1G>A | 38 |
| 16 |  | c.55C>T | 30 |
| 17 |  | c.55C>T | 20 |
| 18 |  | Deletion of entire exon 2 | 33 |
| 19 |  | c.205-2_205-1delAGinsT | 32 |
| 20 |  | c.353_354insA | 63 |
| 21 |  | c.475C>T | 56 |
| 22 |  | c.622G>T | 42 |
| 23 | *CCM3* | c.350_351insT | 8 |

**Supplemental Table 1.** Age at referral to genetic testing of 23 probands carrying a *CCM* mutation published by Stahl et al*.* (2008). The five mutation-negative probands of this series are not included.
